# A MicroRNA Gene Panel Predicts the Vaginal Microbiota Composition

**DOI:** 10.1128/mSystems.00175-21

**Published:** 2021-05-04

**Authors:** Liqin Cheng, Dominika Kaźmierczak, Johanna Norenhag, Marica Hamsten, Emma Fransson, Ina Schuppe-Koistinen, Matts Olovsson, Lars Engstrand, Per Hydbring, Juan Du

**Affiliations:** a Department of Microbiology, Tumor and Cell Biology, Centre for Translational Microbiome Research (CTMR), Karolinska Institutet, Stockholm, Sweden; b Department of Oncology and Pathology, Karolinska Institutet, Stockholm, Sweden; c Department of Women’s and Children’s Health, Uppsala University, Uppsala, Sweden; d Science for Life Laboratory, Karolinska Institutet, Stockholm, Sweden; University of Connecticut

**Keywords:** vaginal microbiota, *Lactobacillus*-dominated, non-*Lactobacillus*-dominated, microRNAs, human papillomavirus

## Abstract

The vaginal microbiota plays an essential role in vaginal health. The vaginas of many reproductive-age women are dominated by one of the *Lactobacillus* species. However, the vaginas of a large number of women are characterized by the colonization of several other anaerobes. Notably, some women with the non-*Lactobacillus*-dominated vaginal microbiota develop bacterial vaginosis, which has been correlated with sexually transmitted infections and other adverse outcomes. However, interactions and mechanisms linking the vaginal microbiota to host response are still under investigation. There are studies suggesting a link between human microRNAs and gut microbiota, but limited analysis has been carried out on the interplay of microRNAs and vaginal microbiota. In this study, we performed a microRNA expression array profiling on 67 vaginal samples from young Swedish women. MicroRNAs were clustered into distinct groups according to vaginal microbiota composition. Interestingly, 182 microRNAs were significantly elevated in their expression in the non-*Lactobacillus*-dominated community, suggesting an antagonistic relationship between *Lactobacillus* and microRNAs. Of the elevated microRNAs, 10 microRNAs displayed excellent diagnostic potential, visualized by receiver operating characteristics analysis. We further validated our findings in 34 independent samples where expression of top microRNA candidates strongly separated the *Lactobacillus*-dominated community from the non-*Lactobacillus*-dominated community in the vaginal microbiota. Notably, the Lactobacillus crispatus-dominated community showed the most profound differential microRNA expression compared with the non-*Lactobacillus*-dominated community. In conclusion, we demonstrate a strong relationship between the vaginal microbiota and numerous genital microRNAs, which may facilitate a deeper mechanistic interplay in this biological niche.

**IMPORTANCE** Vaginal microbiota is correlated with women’s health, where a non-*Lactobacillus*-dominated community predisposes women to a higher risk of disease, including human papillomavirus (HPV). However, the molecular relationship between the vaginal microbiota and host is largely unexplored. In this study, we investigated a link between the vaginal microbiota and host microRNAs in a group of young women. We uncovered an inverse correlation of the expression of microRNAs with the abundance of *Lactobacillus* species in the vaginal microbiota. Particularly, the expression of microRNA miR-23a-3p and miR-130a-3p, displaying significantly elevated levels in non-*Lactobacillus*-dominated communities, predicted the bacterial composition of the vaginal microbiota in an independent validation group. Since targeting of microRNAs is explored in the clinical setting, our results warrant investigation of whether microRNA modulation could be used for treating vaginosis recurrence and vaginosis-related diseases. Conversely, commensal bacteria could be used for treating diseases with microRNA aberrations.

## INTRODUCTION

The human gut microbiota plays an essential role in gut homeostasis. Gut dysbiosis is functionally connected to both intestinal and “non-intestinal diseases” such as cancer, diabetes, and various neurological disorders (1–6). Similarly, the vaginal microbiota plays an essential role in vaginal health (7, 8). The vaginas of many reproductive-age women are dominated by one of the *Lactobacillus* species, including L. crispatus, L. gasseri, L. jensenii, and L. iners (9, 10). The vaginas of a large number of women are also characterized by the colonization of several other anaerobes, such as Gardnerella vaginalis, Atopobium vaginae, and Prevotella bivia, together with a great reduction of the *Lactobacillus* populations (9, 10). Notably, some of the women with the non-*Lactobacillus*-dominated vaginal microbiota develop bacterial vaginosis, which has also been correlated with sexually transmitted infections such as human immunodeficiency virus (HIV) and human papillomavirus (HPV) (11–13). Non-*Lactobacillus*-dominated vaginal microbiota has also been associated with other adverse outcomes, including preterm birth, low birth weight, and potentially affect the microbiota of offspring (14–16). However, despite the critical role of vaginal microbiota, studies linking the vaginal microbiota to host response are still under investigation.

MicroRNAs (miRNAs) belong to a class of small noncoding RNAs, normally 20 to 22 nucleotides in length, with a central role in the posttranscriptional control of gene expression. miRNAs present in body fluids differ in their expression when profiled from healthy individuals compared to patients. Genetic and epigenetic alterations of specific miRNAs have been demonstrated to have causative links to disease development, and during recent years, miRNA therapies have come under development for a wide variety of diseases (17). There is an increasing body of evidence suggesting that miRNAs have crucial functions in responses to pathogenic infections (18–20). Furthermore, specific miRNAs have been demonstrated to be repressed by pathogens, and expression of such miRNAs may be restored by commensal bacteria (21, 22).

Studies performed in mice have suggested a link between the gut microbiota and miRNAs (18, 23). The dominating message is that the microbiota modulates miRNA expression, but the opposite has also been proposed, indicating a role of miRNAs shaping the gut microbiota (24–28). However, there are limited data on the correlation between the gut microbiota and miRNAs in humans. The alteration of miRNA expression has been tied to the gut microbiota in colorectal cancer patients (29). Other studies have suggested complex interactions between the composition of bacteria, host miRNAs, and colorectal cancer development (30, 31). A recent study showed the interplay between the gut microbiota and miRNAs in patients with Crohn’s disease (32).

All of the studies above suggest an active participation of the gut microbiota in reprogramming host gene expression, but there is a substantially smaller amount of information on the relationship between the vaginal microbiota and miRNAs. Thus, this study aims to investigate the correlation between the vaginal microbiota and human miRNAs. Vaginal swab samples were collected from 67 young women between 14 and 29 years of age. Human miRNA profiles were analyzed and compared according to vaginal microbiota composition. Furthermore, two differentially expressed miRNAs emerging from the test samples were validated in 34 independent samples.

## RESULTS

### An overall higher expression of miRNA profiles was observed in non*-Lactobacillus*-dominated samples.

**FIG 1:**
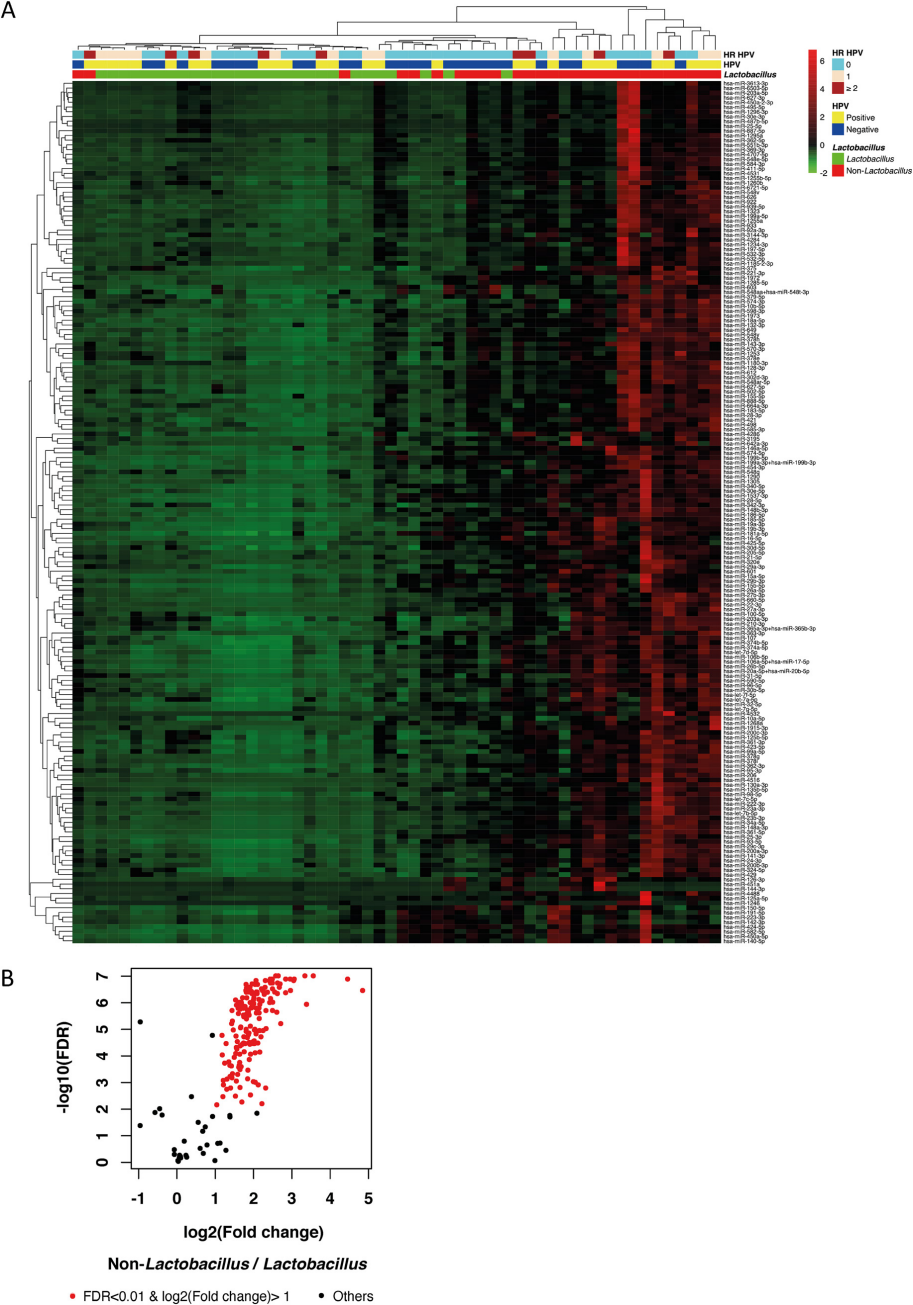
Differential analysis of miRNA expression. (A) Hierarchical clustering analysis of miRNA expression in *Lactobacillus*-dominated samples versus non-*Lactobacillus-*dominated samples, human papillomavirus (HPV)-positive samples versus HPV-negative samples, and different status considering only high-risk (HR) HPV types. Color scale depicts standard deviations from the mean of each row. hsa, Homo sapiens. (B) Volcano plot of downregulated and upregulated miRNAs in *Lactobacillus*-dominated samples compared with non-*Lactobacillus-*dominated samples. The significantly changed miRNAs (>2-fold; false discovery rate: FDR < 0.01) are marked in red color.

In order to address the association of miRNA profiles with HPV status and the vaginal microbiota composition, we took 67 samples from young women in Sweden, extracted RNA, and carried out a systematic miRNA expression profiling. The HPV status and vaginal microbiota composition were obtained from a previously described study (11). Using hierarchical clustering analysis, we uncovered a broad increase in miRNA expression in non-*Lactobacillus-*dominated samples compared with *Lactobacillus*-dominated samples (Fig. 1A). In total, 182 miRNAs displayed significantly higher expression (>2-fold; false discovery rate [FDR] of <0.01) in non*-Lactobacillus*-dominated samples compared with *Lactobacillus-*dominated samples (Fig. 1B; see Table S1 in the supplemental material). In contrast to *Lactobacillus*, HPV status showed no significant impact on miRNA expression (Fig. S1). No miRNAs displayed differential expression (>2-fold; *P* < 0.05) when HPV-positive and HPV-negative samples (see Fig. S1 in the supplemental material) were compared.

10.1128/mSystems.00175-21.1 FIG S1 (A) Volcano plot of downregulated and upregulated miRNAs in HPV-positive samples compared with HPV-negative samples. (B) Volcano plot of downregulated and upregulated miRNAs in samples infected with more than one HR HPV type compared with HPV-negative samples. HR HPV, high-risk HPV. Download FIG S1, TIF file, 0.5 MB. Copyright © 2021 Cheng et al. 2021 Cheng et al. https://creativecommons.org/licenses/by/4.0/ This content is distributed under the terms of the Creative Commons Attribution 4.0 International license.

10.1128/mSystems.00175-21.6 TABLE S1 Differential miRNA gene expression list. Download Table S1, XLSX file, 0.1 MB. Copyright © 2021 Cheng et al. 2021 Cheng et al. https://creativecommons.org/licenses/by/4.0/ This content is distributed under the terms of the Creative Commons Attribution 4.0 International license.

More than 85% (23 out of 27) of HPV-positive women were positive for at least one high-risk (HR) HPV. Since our former data indicated that non-*Lactobacillus-*dominated women had a higher risk of infection with HR HPV, we performed the analysis by further comparing the HR HPV-positive group with the HPV-negative group. Similar results were obtained with no HPV samples clustering according to miRNA expression (Fig. 1A). By further dividing the samples into L. crispatus*-*dominated and L. iners-dominated subgroups, the majority of *L. iners*-dominated samples were mixed with L. crispatus*-*dominated samples but clearly separated from non-*Lactobacillus-*dominated samples (Fig. S2). Our data suggest that the miRNA landscape in vaginal tracts is associated with microbiota composition without the influence of HPV status.

10.1128/mSystems.00175-21.2 FIG S2 Differential analysis of miRNA expression. The heatmap corresponds to the hierarchical clustering analysis of miRNA expression in L. crispatus- or *L. iners*-dominated samples and non-*Lactobacillus*-dominated samples, HPV-positive samples versus HPV-negative samples, and different status considering only high-risk (HR) HPV types. Color scale depicts standard deviations from the mean of each row. Download FIG S2, TIF file, 2.8 MB. Copyright © 2021 Cheng et al. 2021 Cheng et al. https://creativecommons.org/licenses/by/4.0/ This content is distributed under the terms of the Creative Commons Attribution 4.0 International license.

### MicroRNA gene signature distinguishes *Lactobacillus*-dominated and non-*Lactobacillus*-dominated vaginal microbiota.

**FIG 2:**
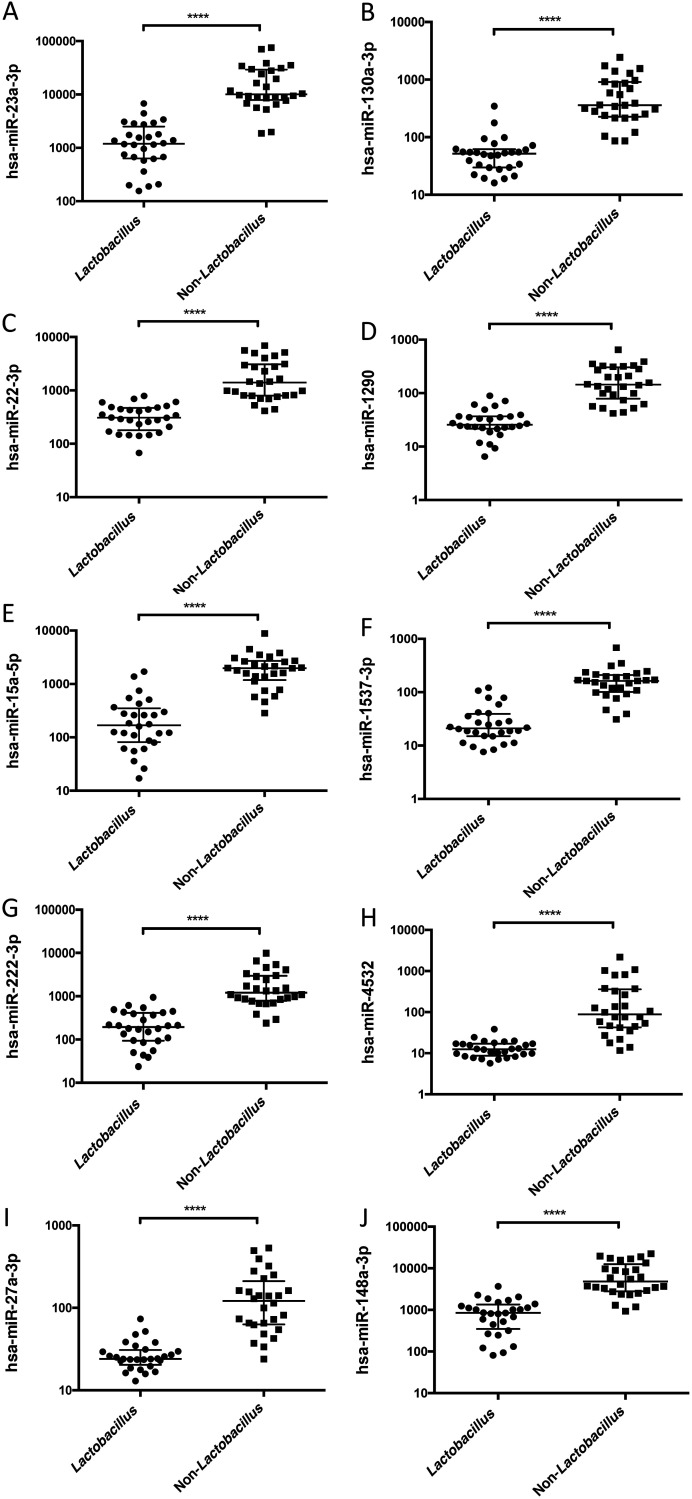
(A to J) Scatterplots of the 10 most differentially expressed miRNAs in *Lactobacillus*-dominated samples versus non-*Lactobacillus-*dominated samples. Data are presented as medians with interquartile ranges (error bars) on a log_10_ scale of normalized expression counts. ****, *P* value of <0.0001.

To further investigate the correlation between miRNA and microbiota composition, we looked into the 10 most differentially expressed miRNAs in non-*Lactobacillus*-dominated samples compared with *Lactobacillus-*dominated samples. All miRNAs were substantially elevated in non-*Lactobacillus*-dominated samples (*P* < 0.0001) (Fig. 2A to J). Dividing the samples in the *Lactobacillus*-dominated group further into L. crispatus*-*dominated and *L. iners*-dominated subgroups did not alter the outcome. miRNA expression was significantly lower in both L. crispatus*-*dominated and *L. iners*-dominated samples compared with non-*Lactobacillus-*dominated samples (Fig. S3A to J). Differential expression of miRNAs between L. crispatus*-*dominated and *L. iners*-dominated subgroups did not reach statistical significance (Fig. S3A to J).

10.1128/mSystems.00175-21.3 FIG S3 (A to J) Scatterplots of the 10 most differentially expressed miRNAs among L. crispatus- or *L. iners*-dominated samples and non-*Lactobacillus*-dominated samples. Data are presented as medians with interquartile ranges on a log_10_ scale of normalized expression counts. ****, *P* value < 0.0001; ***, *P* value < 0.001; n.s., not significant (*P* value of > 0.05). Download FIG S3, TIF file, 0.7 MB. Copyright © 2021 Cheng et al. 2021 Cheng et al. https://creativecommons.org/licenses/by/4.0/ This content is distributed under the terms of the Creative Commons Attribution 4.0 International license.

**FIG 3:**
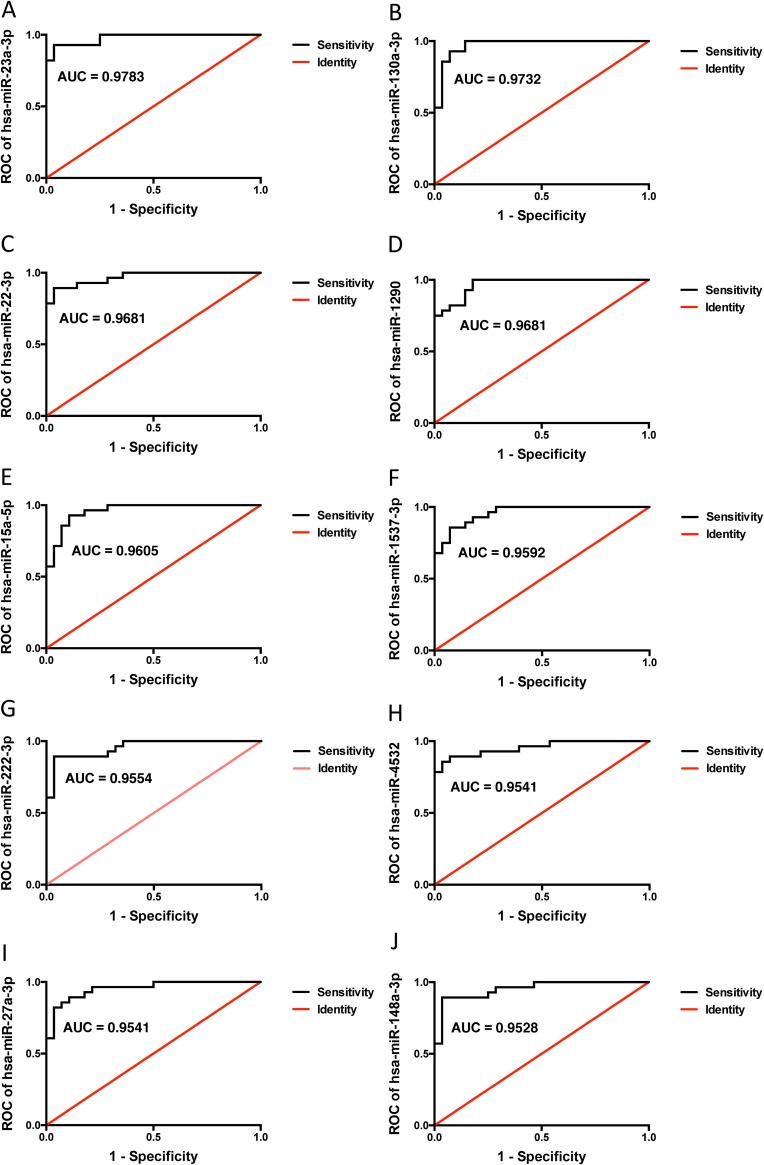
(A to J) Receiver operating characteristics (ROC) curves of the 10 most differentially expressed miRNAs in *Lactobacillus*-dominated samples versus non-*Lactobacillus-*dominated samples. Values for area under curve (AUC) are displayed in each plot, as a measure of the diagnostic potential of each miRNA.

We continued to analyze the diagnostic ability of the most differentially expressed miRNAs. Among these, 10 miRNAs displayed excellent prediction scores and performed specificity over 95% in receiver operating characteristics (ROC) analysis according to an area under curve (AUC) calculation (Fig. 3A to J). miR-23a-3p and miR-130a-3p emerged as the best performing miRNAs, reaching above 97% prediction accuracy (AUC = 0.9783 and 0.9732, respectively) (Fig. 3A and B).

### Prediction of miRNA signature target genes and pathways.

**FIG 4:**
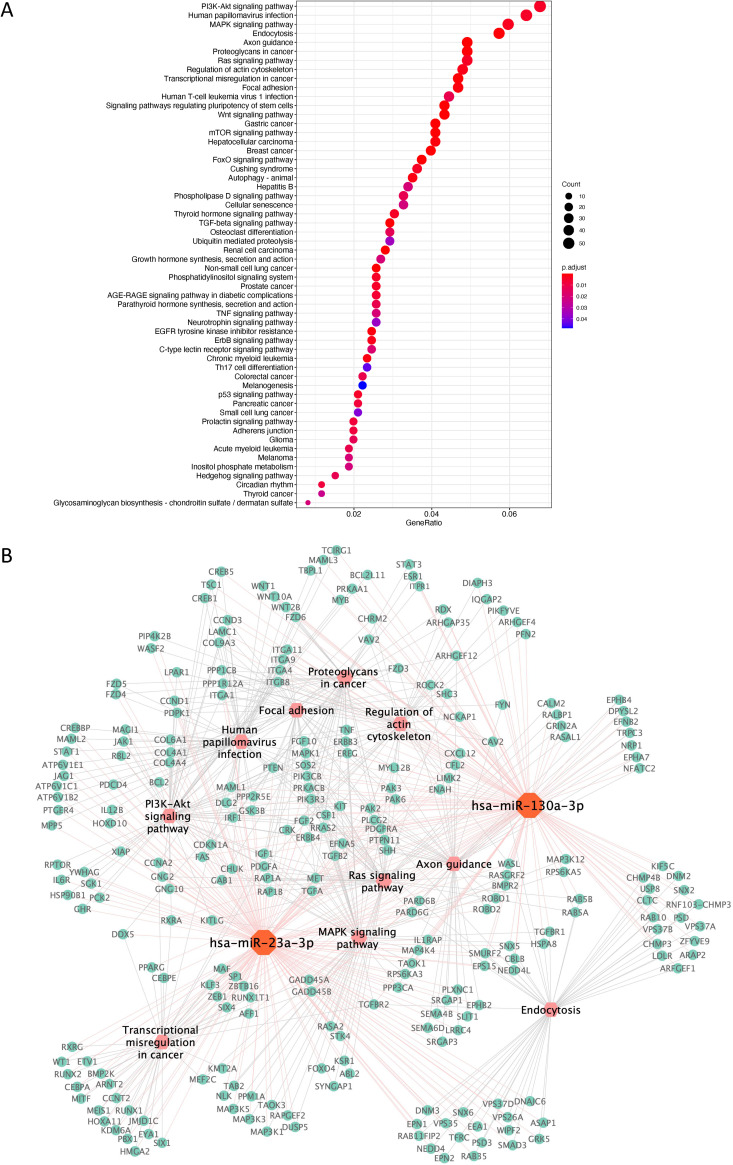
Kyoto Encyclopedia of Genes and Genomes (KEGG) pathway enrichment analysis of miR-23a-3p and miR-130a-3p predicted target genes. (A) Dot plot of the KEGG pathways ranked according to the numbers of miR-23a-3p and miR-130a-3p predicted target genes (TargetScan 7.2) in each pathway. The color of each dot reflects the level of statistical significance. The size of the dots reflects the number of miRNA-predicted target genes in each pathway. (B) The interaction network of miR-23a-3p and miR-130a-3p, their respective predicted target genes (TargetScan 7.2), and the top 10 KEGG pathways from panel A.

We combined miR-23a-3p and miR-130a-3p, which were the top two hits according to differential expression in *Lactobacillus*-dominated samples and non-*Lactobacillus-*dominated samples and analyzed predicted target genes with miRTarBase 7.0. We found 2,144 potential miRNA target genes. A Kyoto Encyclopedia of Genes and Genomes (KEGG) pathway enrichment analysis of these genes found 56 related pathways. As shown in Fig. 4, all top significantly changed pathways are involved in cancer development or infections. Pathways that promote cell proliferation, including the phosphatidylinositol 3-kinase (PI3K)−Akt signaling pathway, the mitogen-activated protein kinase (MAPK) signal pathway, and the Ras signaling pathway, were all among top statistically significant pathways. Furthermore, proteoglycans and transcriptional misregulation pathways, which play an important role in cancer development, and pathways such as endocytosis, actin cytoskeleton regulation, and focal adhesion were also enriched among the predicted target genes of the top two differentially expressed miRNAs (Fig. 4A).

All of these pathways were located at the center node of the network. The miRNA-mRNA-KEGG pathway network of the top 10 pathways was shown in Fig. 4B. The genes that are targeted by miR-23a-3p and miR-130a-3p, which serve as the foundation for the pathways, are listed in the network (Fig. 4B). We also performed a KEGG pathway analysis for the top 10 miRNAs, with a specificity over 95% in ROC analysis. Similar pathways, including the PI3K-Akt signaling pathway, the MAPK signal pathway, and HPV infection were uncovered as the top enriched pathways (Fig. S4).

10.1128/mSystems.00175-21.4 FIG S4 Kyoto Encyclopedia of Genes and Genomes (KEGG) pathway enrichment analysis of predicted target genes from the 10 most differentially expressed miRNAs in *Lactobacillus*-dominated samples versus non-*Lactobacillus-*dominated samples. The dot plot shows the top 50 KEGG pathways ranked according to the number of genes mapped to each pathway. The color of each dot reflects the level of statistical significance. The size of the dots reflects the number of miRNA-predicted target genes in each pathway. Download FIG S4, TIF file, 1.8 MB. Copyright © 2021 Cheng et al. 2021 Cheng et al. https://creativecommons.org/licenses/by/4.0/ This content is distributed under the terms of the Creative Commons Attribution 4.0 International license.

### Validation samples confirmed the prediction of the vaginal microbiota to be dictated by miRNA expression.

**FIG 5:**
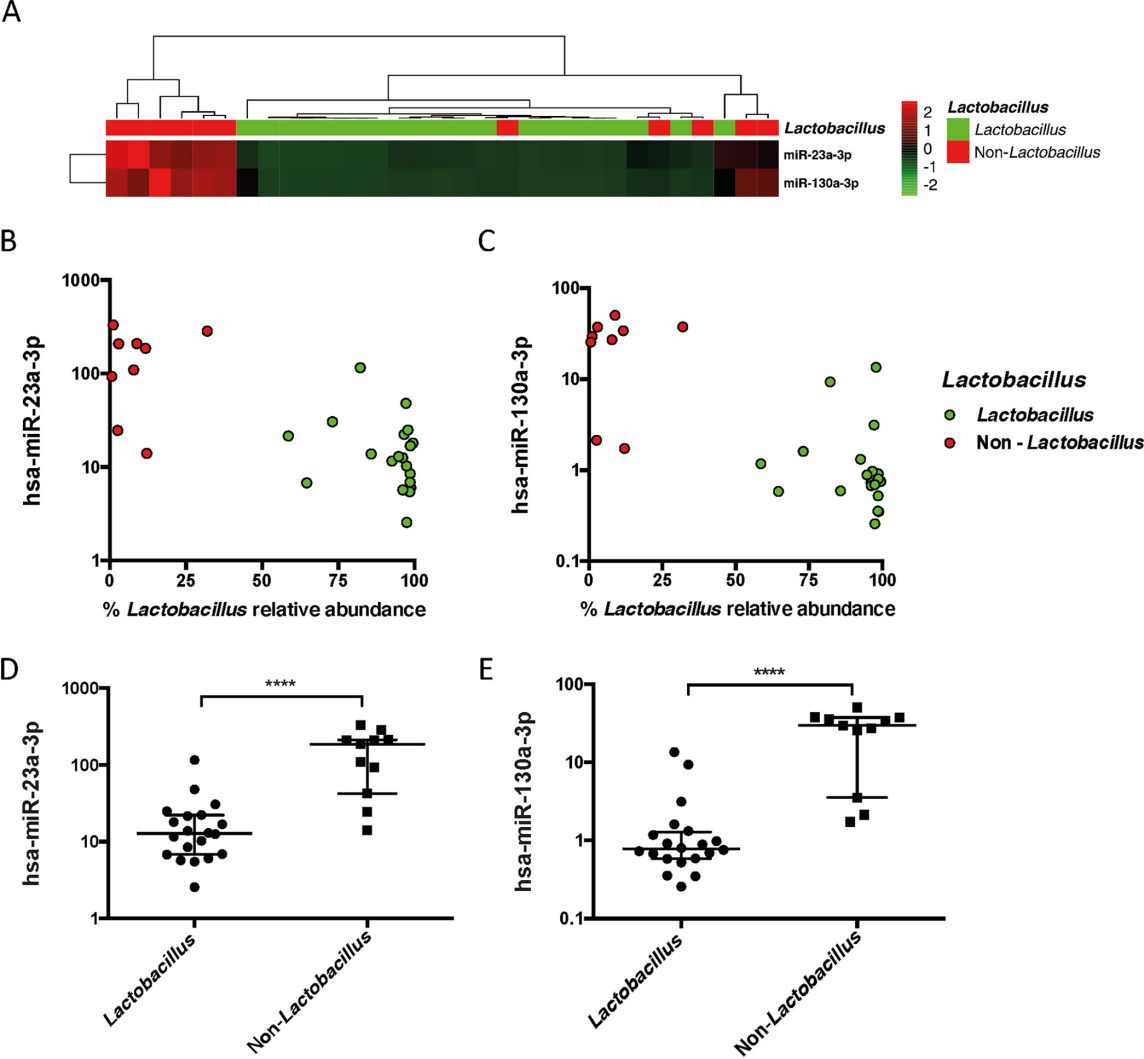
Validation of miR-23a-3p and miR-130a-3p expression in *Lactobacillus*-dominated samples versus non-*Lactobacillus-*dominated samples from a distinct group of vaginal samples. (A) Hierarchical clustering of miR-23a-3p and miR-130a-3p expression in *Lactobacillus*-dominated samples versus non-*Lactobacillus-*dominated samples. The color scale depicts standard deviations from the mean of each row. (B and C) Scatterplots displaying the association between miR-23a-3p and miR-130a-3p expression with *Lactobacillus* species abundance. (D and E) Comparison of miR-23a-3p and miR-130a-3p expression in *Lactobacillus*-dominated samples versus non-*Lactobacillus-*dominated samples. Data are presented as median with interquartile range on a log_10_ scale. *y*-axis values depict the percentage of expression in relation to U6 snRNA. ****, *P* value of <0.0001.

To validate our finding, we analyzed an independent group of 34 samples by reverse transcription-quantitative PCR (RT-qPCR). Nearly all samples clustered into two distinct groups of *Lactobacillus*-dominated versus non-*Lactobacillus*-dominated groups by the expression level of miR-23a-3p and miR-130a-3p (Fig. 5A). As shown in Fig. 5B and C, the *Lactobacillus* species relative abundance is increasing with decreased expression of miR-23a-3p and miR-130a-3p. The *Lactobacillus*-dominated and non-*Lactobacillus*-dominated samples were separated clearly based on miR-23a-3p and miR-130a-3p expression (Fig. 5B and C). When *Lactobacillus*-dominated and non-*Lactobacillus*-dominated groups were divided, a significantly higher level was observed in the non-*Lactobacillus*-dominated group compared with the *Lactobacillus*-dominated group for both miR-23a-3p (*P* < 0.0001) and miR-130a-3p (*P* < 0.0001) (Fig. 5D and E).

Further division by L. crispatus-dominated and *L. iners*-dominated subgroups demonstrated L. crispatus-dominated samples with the lowest expression of miR-23a-3p and miR-130a-3p, and *L. iners*-dominated samples with moderate expression of miR-23a-3p and miR-130a-3p, compared with the highest expression level in non-*Lactobacillus*-dominated samples (Fig. S5A and B). Similar to the tested group, both the L. crispatus-dominated and *L. iners*-dominated subgroups had significantly lower expression of miR-23a-3p and miR-130a-3p compared with non-*Lactobacillus*-dominated samples in the validation group. Differential expression between L. crispatus-dominated and *L. iners*-dominated samples did not reach statistical significance (Fig. S5C and D).

10.1128/mSystems.00175-21.5 FIG S5 Validation of miR-23a-3p and miR-130a-3p in L. crispatus-dominated samples, *L. iners*-dominated samples versus non-*Lactobacillus-*dominated samples from a distinct group of vaginal samples. (A and B) Scatterplots displaying the association between miR-23a-3p and miR-130a-3p expression with *Lactobacillus* sp. abundance. (C and D) Scatterplots of miR-23a-3p and miR-130a-3p expression in L. crispatus- or *L. iners*-dominated samples and non-*Lactobacillus*-dominated samples. Data are presented as medians with interquartile ranges on a log_10_ scale. *y*-axis values depict the percentage of expression in relation to U6 snRNA. ****, *P* value < 0.0001; *, *P* value < 0.05; n.s., not significant (*P* value > 0.05). Download FIG S5, TIF file, 0.5 MB. Copyright © 2021 Cheng et al. 2021 Cheng et al. https://creativecommons.org/licenses/by/4.0/ This content is distributed under the terms of the Creative Commons Attribution 4.0 International license.

## DISCUSSION

In this study, we observed a higher miRNA expression in non-*Lactobacillus*-dominated vaginal samples compared with *Lactobacillus*-dominated vaginal samples. Differential expression was particularly obvious when comparing non-*Lactobacillus*-dominated samples with L. crispatus-dominated samples. We propose a panel of differentially expressed miRNAs that distinguishes *Lactobacillus*-dominated from non-*Lactobacillus*-dominated communities. Notably, the 10 miRNAs displayed excellent diagnostic prediction values (AUC of >0.95) for vaginal microbiota. Furthermore, the top two differentially expressed miRNAs were validated in a separate group of 34 samples by RT-qPCR with a highly significant prediction rate.

This study establishes a link between human miRNAs and vaginal microbiota which may partly explain how microbes influence the host or vice versa. We observed a substantial increase in expression of numerous miRNAs in the non-*Lactobacillus*-dominated group. A previous report showed that miR-193b, miR-203b, miR-320b-1, miR-223, and miR-183 were found to correlate with the abundance of the *Lactobacillus* species in a study utilizing small RNA transcriptome sequencing (33). The study further suggests that *Lactobacillus* spp. control the cell cycle by a global mechanism, which is in line with our findings of broad expression changes in the miRNA landscape (33). We also observed miR-223 and miR-183 to be significantly altered in our group of samples. However, we did not observe miR-193b, miR-203b, and miR-320b-1 to be differentially expressed, which may be due to differences in sample types or in the methodology for miRNA expression analysis. Another study demonstrated changes in miRNA expression following HPV infection (34). However, we did not find any miRNA differences between HPV-positive and HPV-negative individuals in our study, which rules out a potential influence of HPV status on our microbiota results.

In addition, the study of a single pathogen and its linkage to alterations of miRNAs also provides support for the interaction between host and bacteria (19). However, to our knowledge, miR-23a-3p and miR-130a-3p have not been studied in relation to vaginal microbes, possibly since most previous studies were carried out on gut microbiota or pathogens. miR-23a-3p has been related to cancers, cervical shortening, and preterm birth, diseases which are linked to non-*Lactobacillus*-dominated vaginal microbiota (35–38). There is also literature suggesting that miR-23a-3p may regulate Notch signaling pathway, with a higher expression in cervical cancer patients (36, 37). miR-130a-3p has also been shown to be involved in the proliferation and apoptosis of cancer cells and to assist cancer metastasis and invasion (39). Most importantly, through signaling pathways, including Wnt and phosphatase and tensin homolog (PTEN), miR-130a-3p could affect drug susceptibility and has been proposed as a new therapeutic target for cancers (39). Furthermore, miR-130a-3p has been shown as a regulator of Dicer expression in cervical cancer and was significantly associated with poor disease-free survival (40).

From our network analysis on both the top two and top ten miRNAs, the most strongly related pathways were the PI3K-Akt signaling, MAPK signaling, and Ras signaling pathways. These are pathways containing essential players for initiating cell cycle progression. Somatic mutations in these pathways often occur in many tumors (41, 42). Moreover, the proteoglycans and transcriptional misregulation pathways are also of importance in cancer development (43, 44). A higher involvement of these pathways among the non-*Lactobacillus*-dominated group suggests the possibility that the non-*Lactobacillus* species in the vaginal tract may lead to the promotion of carcinogenesis. This is supported by published data that non-*Lactobacillus*-dominated vaginal microbiota was correlated with cervical intraepithelial neoplasia and cervical cancer (45). Moreover, alteration of endocytosis, actin cytoskeleton regulation, and focal adhesion in the pathways could also benefit non-*Lactobacillus* bacteria to establish their niches in the vaginal tract and potentially to fight against host defense and colonization resistance from other bacteria. However, our network analysis is solely based on *in silico* prediction of miRNA targets and does not necessarily reflect a true biological outcome of the vaginal microbiota.

Our study provides support of the interplay between non-*Lactobacillus* species and miRNAs. However, insight into the mechanism of how the vaginal microbiota is linked to human miRNAs needs further laboratory efforts. Most of our current knowledge between microbiota and miRNAs is obtained from miRNA profiles in germfree mice versus conventional mice. One possible mechanism could be that microbes interact with cell surface receptors and influence the cell cycle pathway which further affects miRNA expression (33). Interestingly, numerous miRNAs are enriched in targeting the cell cycle machinery, and such miRNAs may be specifically blocked by the interaction of microbes with the host (46). Microbes could also interact with the host by triggering or blocking host immune response (21, 31, 47, 48). One study suggested that commensal microbiota-induced miRNAs could affect intestinal epithelial permeability (25). Furthermore, the interaction might be indirect through metabolites or proteins secreted by microbes, or conversely, that host miRNAs favor specific microbes and thus change the trajectory of bacterial composition (28). A major limitation of our study is that we do not know the biological mechanism contributing to the increased expression of numerous miRNAs. The differential expression of multiple miRNAs could be a passenger event of non-*Lactobacillus* infection, such as an altered cellular landscape due to infiltrating components from the immune system. Future investigations of the full transcriptome in this type of samples, or cellular analysis on freshly collected samples, may help to decipher the cellular composition in the *Lactobacillus*-dominated versus non-*Lactobacillus*-dominated groups. Another limitation is the scarcity of clinical data, including missing data on vaginal pH and sexual activity.

It is well-known that the vaginal microbiota has an important role in the fight against sexually transmitted viruses and non-*Lactobacillus*-dominated communities have been linked to a higher risk of cervical cancer lesions (11, 33, 45, 49, 50). Our study provides a hint of broad alteration of miRNAs related to non-*Lactobacillus*-dominated vaginal microbiota compared with *Lactobacillus*-dominated vaginal microbiota. Future studies should investigate whether alterations of these miRNAs reflect a causality link between the vaginal microbiota and miRNAs rather than a passenger event. A better understanding of the interplay between vaginal microbiota and host response would aid the development of novel therapeutics to protect women from infection and diseases.

## MATERIALS AND METHODS

### Study population.

Previously described young women between 14 and 29 years from a youth clinic (*n* = 32) and cervical screening (*n* = 35) were selected. Vaginal swabs were collected either by clinical staff or self-collected (11). The collection swabs were preserved with 0.8 ml DNA/RNA-shield (Zymo Research, USA) in FluidX tubes (Brooks Life Sciences, USA). Information regarding HPV vaccination status, age, and antibiotic usage within the past 3 months was included with the sample. All participation was voluntary, and written informed consent was obtained before sample collection.

DNA extraction, HPV genotyping, and microbiota sequencing on the V3-V4 regions of the 16S rRNA genes were performed as before (11, 51). Information of HPV status and microbiota composition was obtained from the previous study (11, 51). Out of the 67 samples, 5 were excluded due to antibiotic usage during the past 3 months, and a further 6 were excluded for low sequencing reads (operational taxonomic unit [OTU] < 20,000). In the end, 56 samples were included in the final analysis, in which 27 samples were HPV positive and 29 were HPV negative. Vaginal microbiota of 14 samples were dominated by L. crispatus, 14 samples were dominated by L. iners, and 28 samples were non-*Lactobacillus*-dominated samples.

An additional 34 samples from youth clinic were used to validate the prediction association model, in which three samples were excluded in the final analysis due to low sequencing reads. Details of the participants both in the test group and the validation group are listed in Table S2 in the supplemental material. Ethical approval was granted by the Stockholm Regional Ethics Committee and Uppsala Regional Ethics Committee in Sweden to examine the youths without parental consent.

10.1128/mSystems.00175-21.7 TABLE S2 Characteristics of recruited individuals. Download Table S2, TIF file, 0.04 MB. Copyright © 2021 Cheng et al. 2021 Cheng et al. https://creativecommons.org/licenses/by/4.0/ This content is distributed under the terms of the Creative Commons Attribution 4.0 International license.

### MicroRNA extraction and systematic expression assay.

Total RNA (including miRNAs) was isolated from vaginal sample collections using a mirVana miRNA isolation kit (catalog no. AM1560; ThermoFisher, USA) following the manufacturer’s protocol. For miRNA expression analysis, nCounter Human v3 miRNA Expression Assay, interrogating the expression of 798 distinct human miRNAs (NanoString Technologies, USA) was used according to the protocol. Briefly, capture and barcoded target probes were hybridized to 100 ng of native total RNA in 3 μl, followed by immobilization on an image surface that is scanned for fluorescence. The number of bound barcoded target probes dictates the fluorescence raw data for each target. Raw data were normalized using the top 100 most abundantly expressed genes. Finally, only miRNAs with an average normalized count exceeding 50 were subjects for analysis.

### MicroRNA expression analysis.

Comparative analysis between the HPV-positive and -negative samples and between *Lactobacillus*-dominated and non-*Lactobacillus-*dominated samples was carried out using fold change and the Wilcox rank sum test. The Kruskal-Wallis H test was utilized to analyze differential expression in L. crispatus-, *L. iners*-, and non-*Lactobacillus*-dominated samples. The FDR was obtained by adjusting the *P* value using the Benjamini-Hochberg algorithm. The requirements for filtering miRNAs were as follows: (i) miRNAs of the two types of samples with a greater than two-fold change compared with the median; (ii) miRNAs of the two types of samples with a corrected *P* value (FDR) lower than 0.01 for microbiota groups and with an uncorrected *P* value lower than 0.05 for HPV groups. Hierarchical clustering was conducted with the settings of Euclidean distance with complete linkage. Visualization as well as a calculation of statistical significance were generated through R statistical language v. 3.6.3. A heatmap of miRNA expression with annotation of HPV status and microbiota composition was constructed by the “pheatmap” package of R. Expression of the 10 most differentially expressed miRNAs in non-*Lactobacillus*-dominated samples versus *Lactobacillus*-dominated samples was visualized by GraphPad Prism software. AUC-ROC analysis was conducted to analyze the sensitivity and specificity of the 10 most differentially expressed miRNAs and visualized by GraphPad Prism software. Expression of these 10 most differentially expressed miRNAs among L. crispatus-, *L. iners*-, and non-*Lactobacillus*-dominated samples was analyzed by Kruskal-Wallis H test with *post hoc* tests using Dunn's multiple-comparison test and visualized with GraphPad Prism software.

### KEGG pathway analysis.

Predicted target genes of differentially expressed miRNAs were extracted from TargetScanHuman 7.2 (52). The target gene set was subjected to KEGG pathway analysis. The KEGG pathway enrichment of these genes was analyzed and visualized by the clusterProfiler package in R. A *P* value of <0.05 was set as the cutoff criterion for the significant enrichment. Cytoscape (v. 3.8.2) was used to visualize the network of miRNAs, mRNAs, and KEGG pathways.

### MicroRNA RT-qPCR.

For the verification of the top two differentially expressed miRNAs in non-*Lactobacillus*-dominated samples versus *Lactobacillus*-dominated samples, miRNA-specific stem-loop cDNA was synthesized from total RNA, extracted by mirVana miRNA isolation kit (catalog no. AM1560; ThermoFisher, USA), and quantified on a NanoDrop 1000 spectrophotometer, using TaqMan miRNA assays (ThermoFisher, USA). The expression of selected miRNAs, miR-23a-3p and miR-130a-3p, were detected. U6 snRNA was used as a reference gene for normalization according to Δ*C_T_* calculation, and miRNA expression was presented as percentage expression of U6 snRNA. A comparative analysis between *Lactobacillus*-dominated and non-*Lactobacillus*-dominated samples was carried out using fold change and the Wilcox rank sum test. The Kruskal-Wallis H test, followed by a Dunn’s multiple-comparison *post hoc* test, was utilized to analyze differences among L. crispatus*-*, *L. iners-*, and non-*Lactobacillus*-dominated samples. A visualization of differentially expressed miRNAs was carried out using GraphPad Prism software.

### Data availability.

All data generated or analyzed in this study are included in this published article (and the supplemental material).
