# Supplementary figures and images for: A MicroRNA Gene Panel Predicts the Vaginal Microbiota Composition
Source: mSystems. 2021 May 4;6(3):e00175-21. doi: 10.1128/mSystems.00175-21 (PMC8269211; doi:10.1128/mSystems.00175-21)

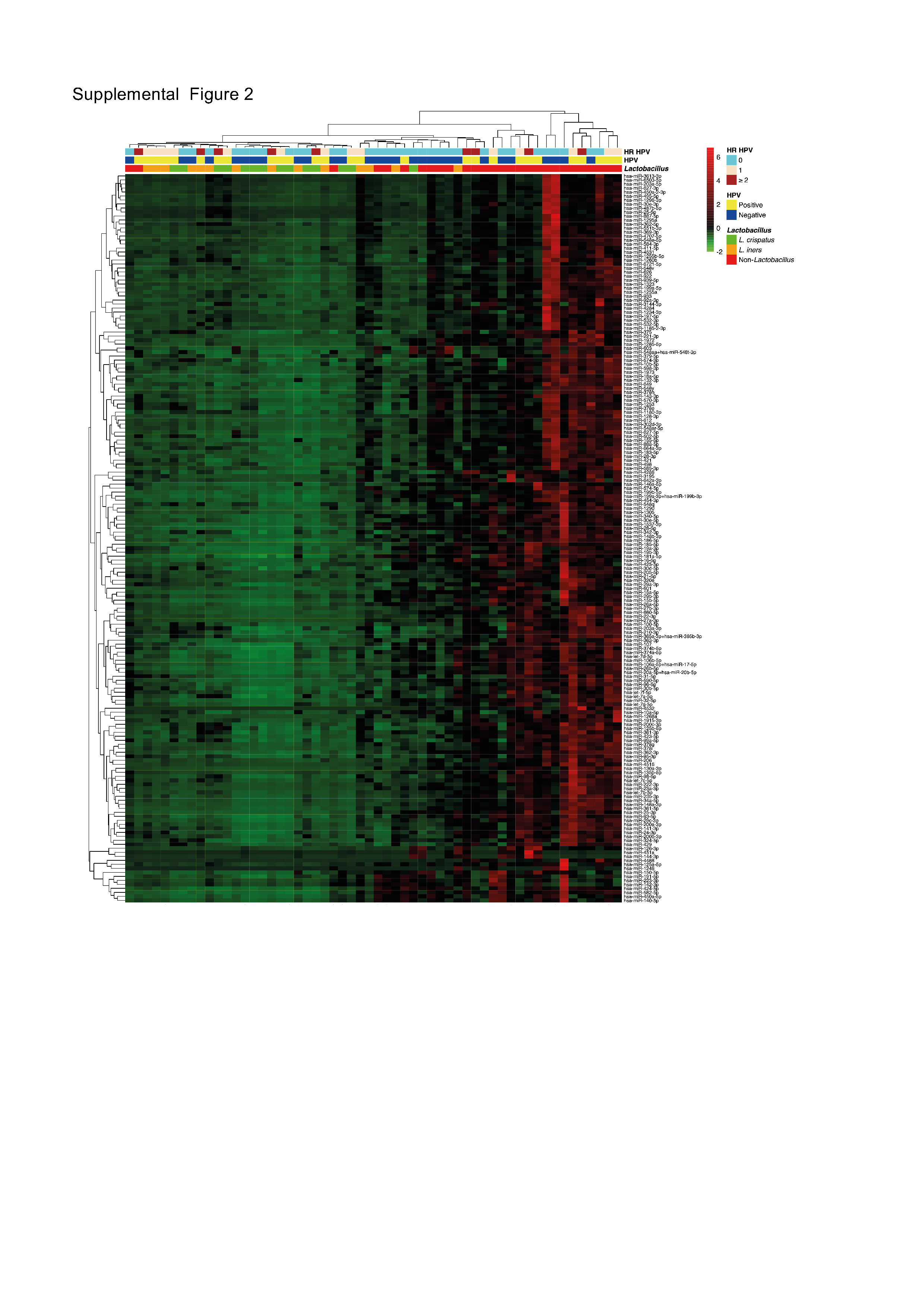

Supplement: FIG S2 [file msystems.00175-21-sf002.tif]

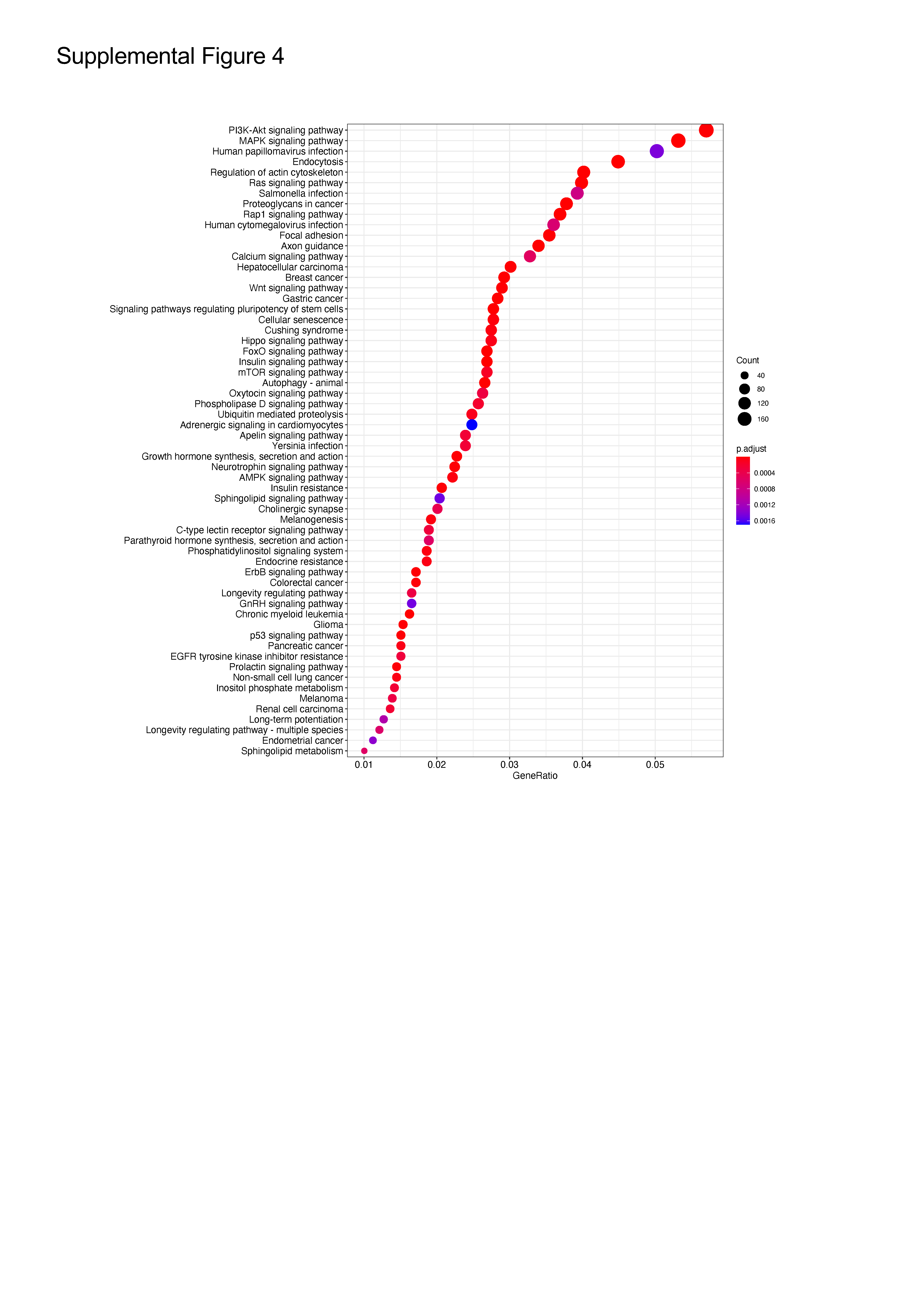

Supplement: FIG S4 [file msystems.00175-21-sf004.tif]

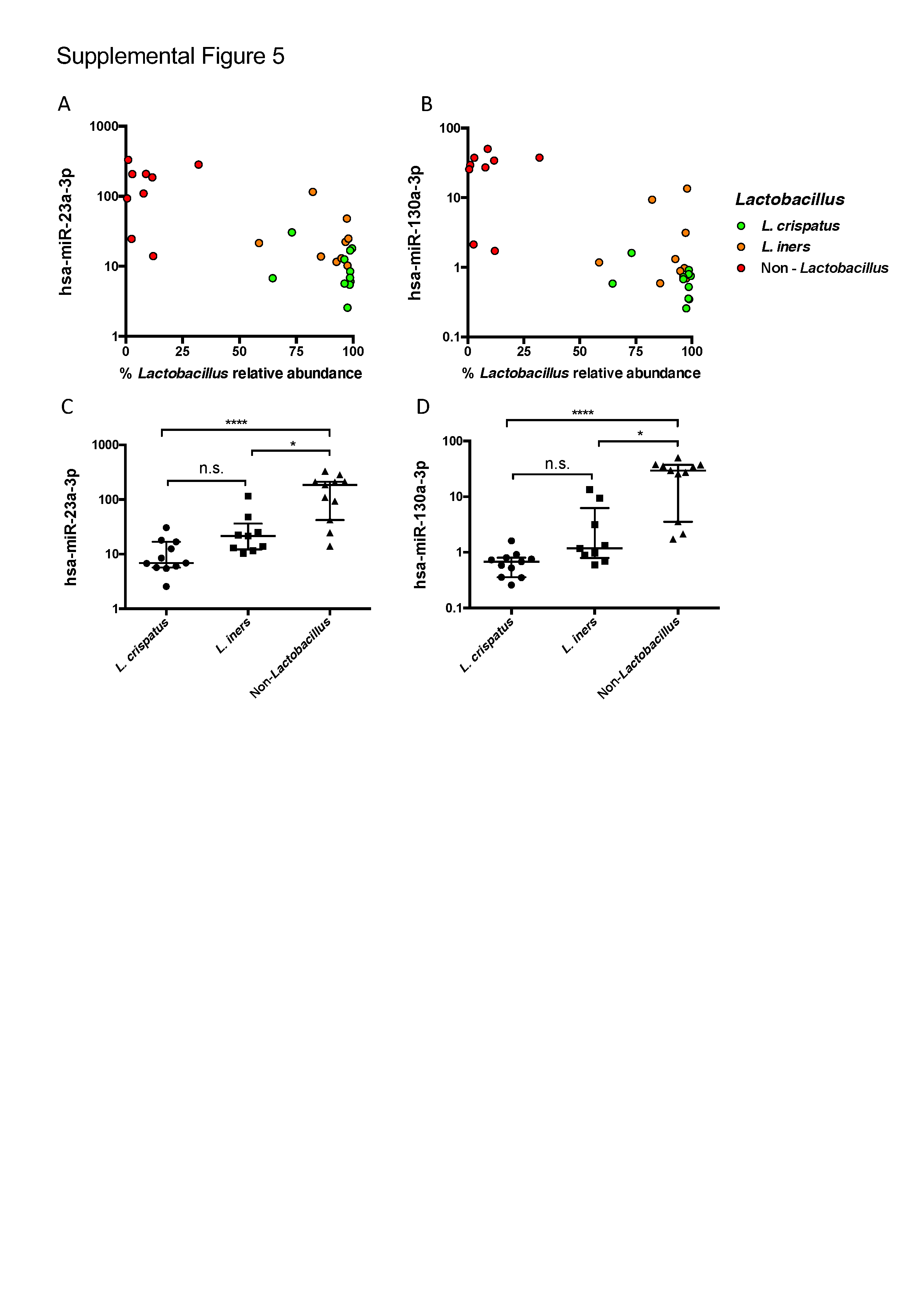

Supplement: FIG S5 [file msystems.00175-21-sf005.tif]
